# Skins Are Simpler Than You Think

**Published:** 1986-02

**Authors:** J. L. Burton

**Affiliations:** Reader in Dermatology, Bristol Royal Infirmary, Bristol, BS2 8HW


### Skins are simpler than you think. Dermatological management for G.P. Trainees

**J. L. Burton BSc, MD, FRCP**
Reader in Dermatology, Bristol Royal Infirmary, Bristol, BS2 8HW

Dermatology is an incredibly simple subject. The fact that there are over 1000 different skin diseases, each of which may appear in several disguises, with a correspondingly confusing nomenclature, is irrelevant, since diagnosis is not necessary for successful therapy.

Dermatological diagnosis is undoubtedly useful for impressing less gifted colleagues who are only to be trusted with mechanistic trivia such as the replacement of heart valves, but its value to the patient is limited. Most patients will accept that they are suffering from 'poikiloderma atrophicans vasculare of Jacobi' just as readily as they would accept the diagnosis of 'pityriasis lichenoides acuta et varioliformis of Mucha-Habermann'.

Alastair Cooke recounts the story of how he once suffered from a very irritating rash around the ankles which completely defeated his own General Practitioner. However, he eventually discovered for himself that the rash was caused by trudging across the salt desert of California for a film he was making, and when he avoided this pastime he was able to report to the G.P. that the rash had fully recovered. 'Thank goodness' said the G.P., 'That means that I shall not have to send you to a dermatologist'. 'Why are you so pleased about that?' asked Cooke. 'Well', said the doctor, 'I share their confusion but not their nomenclature'.

Patients are often unimpressed even by the cleverest diagnosis; what they want to know is the cause, and how to treat it. Patients nowadays are surprisingly well-informed on this subject, and those who read the Guardian will be aware that the aetiological possibilities include living near a nuclear reactor or under an electric power cable, the use of biological washing powder by a lady down the road, knowing a man at work who shook hands with someone thought to have the early stage of AIDS, malpractice by the general practitioner for his personal financial gain, and a severe reaction to a dangerous drug given by a hospital doctor out of idle curiosity, as well as the better-known causes such as lack of vitamins, allergy to almost anything, and 'hot blood'.

Fortunately, there are only three basic types of skin disease; those that make you itch, those that make you smell and those that make you look horrible; and for the trainee G.P., even this classification is unnecessarily complex. For practical purposes skin conditions can be divided into two categories—those that respond to steroid ointments, and those that don't.

Those afflictions that respond to topical steroids present no problem, providing the G.P. can dismiss from his mind the haunting thought that the patient who obtains repeat prescriptions from the receptionist will probably next present with adrenal atrophy, osteoporosis, transparent skin with multiple striae, and the original rash which is now secondarily infected. Possibly the scabies mite which was the original aetiological agent will by that stage also have a moon face and a buffalo hump.

The few rashes that do not respond to topical steroids present more of a problem, but this is easily overcome by the doctor who treats the patient rather than the disease. The first essential is to emphasize to the patient that this is not a trivial condition. Granny may well have said that it is only a sweat rash which should clear up with a bit of ointment from the quack, but the experienced eye of the expert should always discern subtle signs which indicate a poor prognosis. The gloomy outlook should be communicated to the patient after a thoughtful shake of the head whilst sucking air through the teeth. Trainees unfamiliar with this manifestation of professional gravitas should study the style of the garage mechanic who, after a cursory inspection of the damage, sadly informs you 'Won't be much change out of £200, squire'.

The patient should thus be convinced at the outset that the condition is likely to progress rapidly and relentlessly unless it is skilfully treated. Before imparting this information to the patient however, the wise practitioner will establish just how long the condition has already been present, as the advice is much less impressive if the rash has remained completely unchanged for the past 29 years.

When prescribing, remember that any remedy is likely to be successful providing three simple rules are followed:

1. The remedy should be distinctive in colour or smell. Namby-pamby bland white ointments such as hydrocortisone have little chance of success.
2. It should produce a predictable side-effect so that the patient can feel its power. 'Tiger Balm', a harmless counter-irritant, is sold by the ton throughout Asia and the manufacturer is a multi-millionaire.
3. The patient should be told exactly what the remedy is going to do, including the predicted side-effect. This will considerably enhance the placebo response. The doctor-patient relationship will not be improved by failure to mention that the product will bleach the hair and clothing (benzoyl peroxide), make the nails go brown (potassium permanganate), stain the bath permanently (dithranol), turn the cream carpet purple when the treated toddler crawls over it (gentian violet) or cause the patient to smell of garlic (DMSO) or worse.

Provided these simple rules are followed, a successful outcome can be anticipated, since placebos are powerful and the majority of skin diseases are self-limiting.

It will thus be seen that for most patients a diagnosis is unnecessary, but for the more demanding intelligentsia the most useful label is 'chronic benign parapsoriasis'. This term carries a certain dignity which is essential for the middle-class peace of mind, and it is not easy for them to look it up in their Compendium of Home Nursing, particularly as the term is now obsolete.

Some patients will need to be told that this is the worst example of the condition that you have ever seen, so that they can pass this nugget on to their sympathetic friends. Others will need reassurance that theirs is a very mild form of the condition. Hence the importance of well-kept notes so that you can look back and see how they've reacted to such information in the past. Holistic something-or-other, I believe it's called.

In the unlikely event that the patient fails to respond to treatment, special diets in which an ingedient such as red cabbage is rigorously excluded (or alternatively heaped on the plate at every meal), are well worthwhile as a method of passing the time until the expected natural remission occurs. If the patient turns awkward, a more difficult diet such as a milk-and-gluten-free diet should be advised, so that any failure to respond can readily be blamed on the patient's failure to stick to the diet. If that fails, I am afraid you will have to ask the Senior Partner.

